# Supplementary figures and images for: Genome-Wide Identification and Expression Analysis of Bx Involved in Benzoxazinoids Biosynthesis Revealed the Roles of DIMBOA during Early Somatic Embryogenesis in Dimocarpus longan Lour
Source: Plants (Basel). 2024 May 15;13(10):1373. doi: 10.3390/plants13101373 (PMC11125010; doi:10.3390/plants13101373)

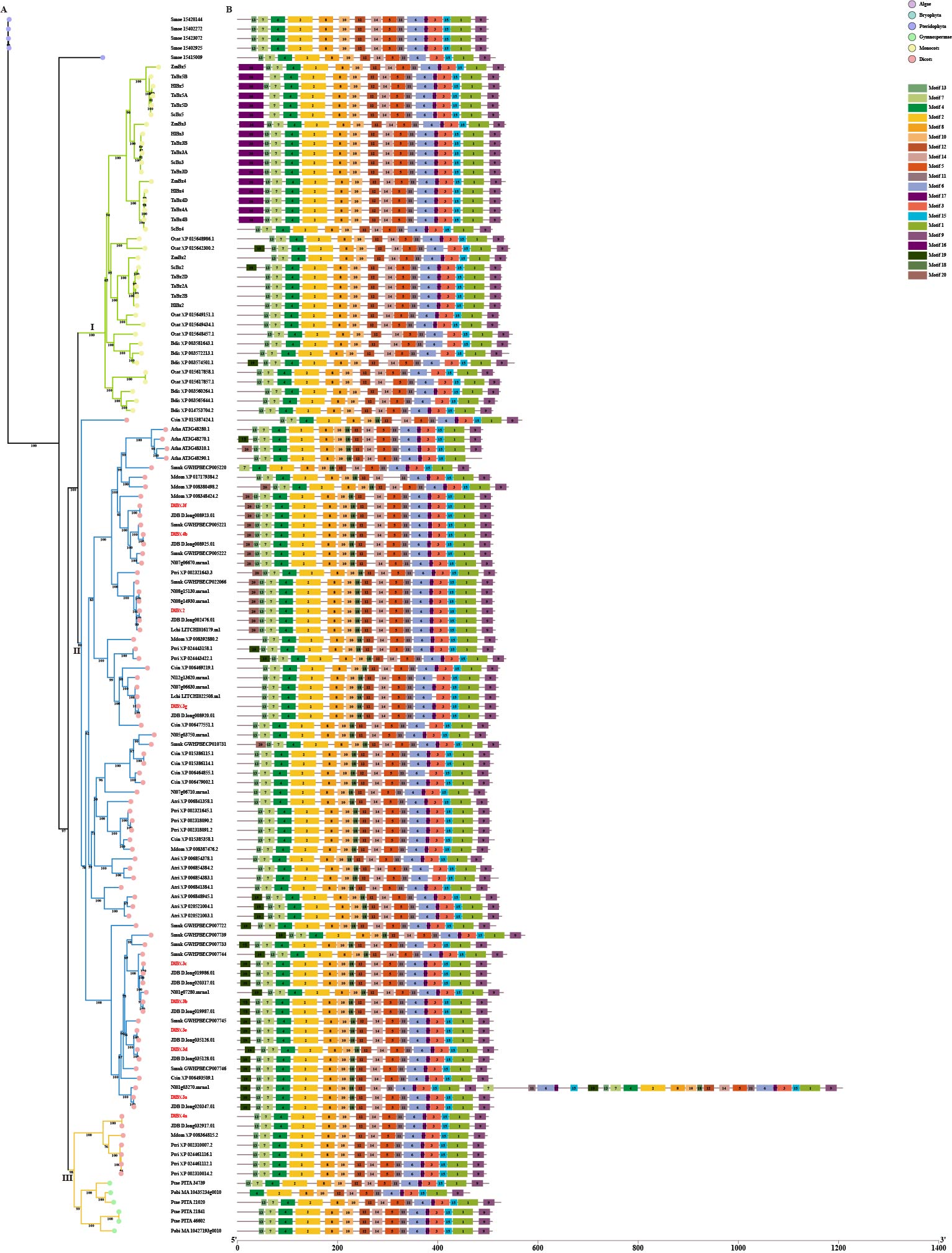

Supplement: Supplementary file 1 [file plants-13-01373-s001.zip › Suppl. Fig S1.jpg]

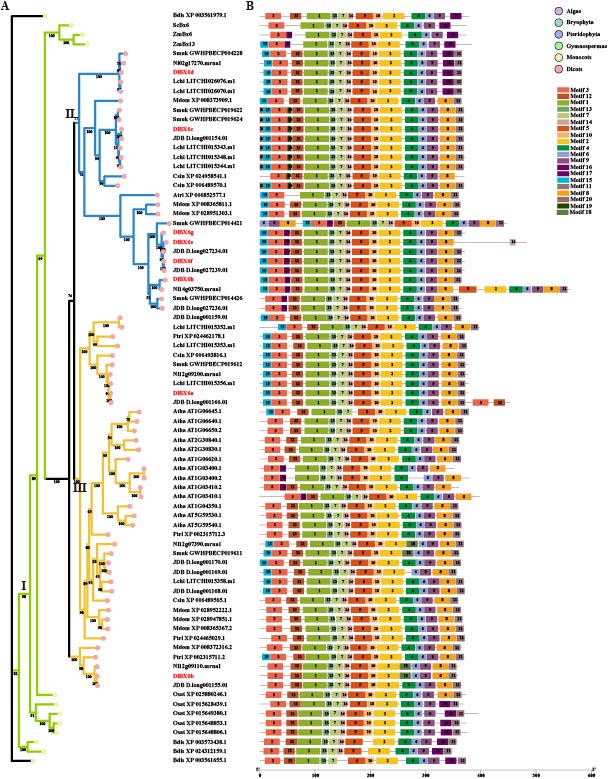

Supplement: Supplementary file 1 [file plants-13-01373-s001.zip › Suppl. Fig S2.jpg]

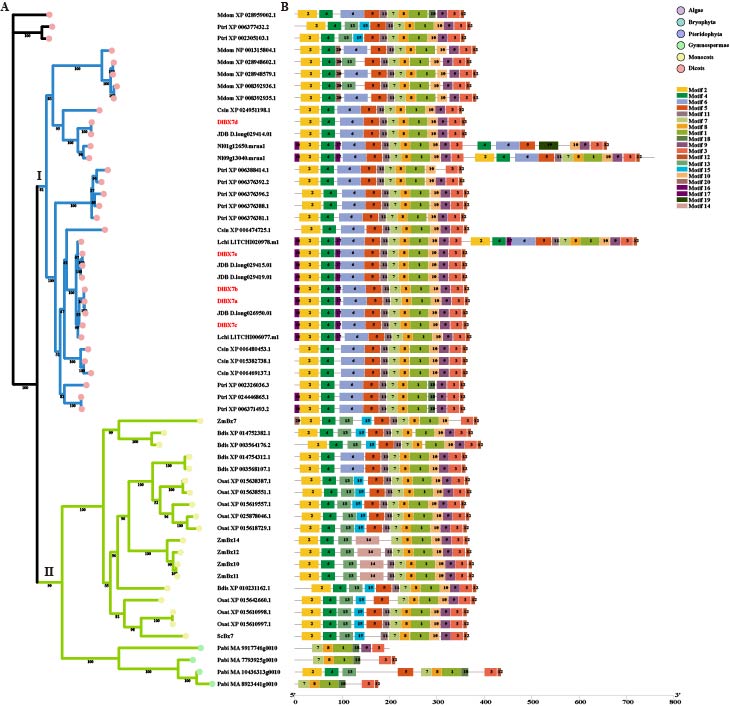

Supplement: Supplementary file 1 [file plants-13-01373-s001.zip › Suppl. Fig S3.jpg]

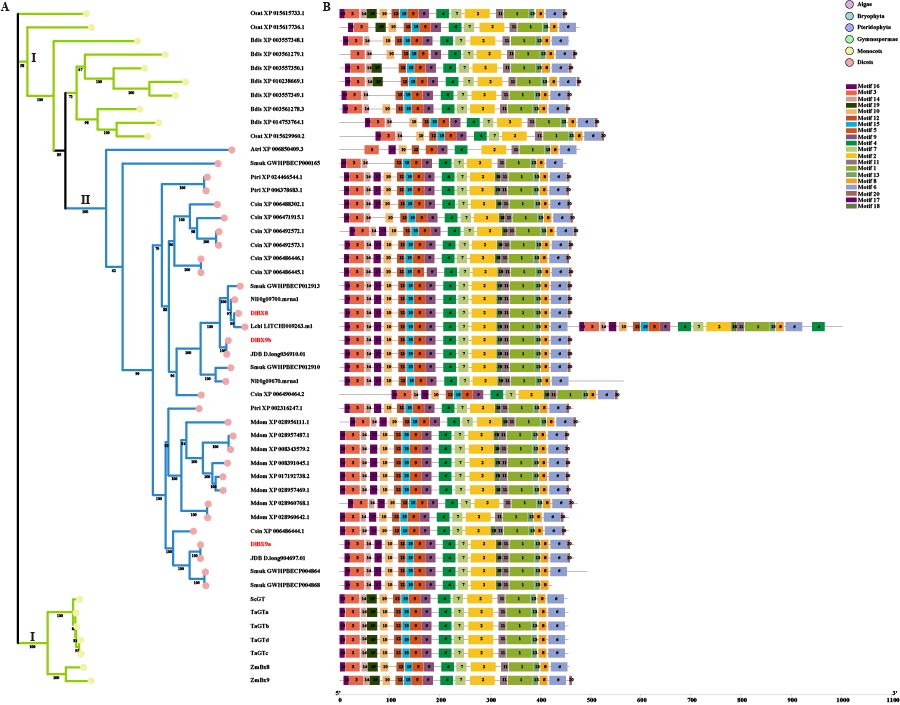

Supplement: Supplementary file 1 [file plants-13-01373-s001.zip › Suppl. Fig S4.jpg]

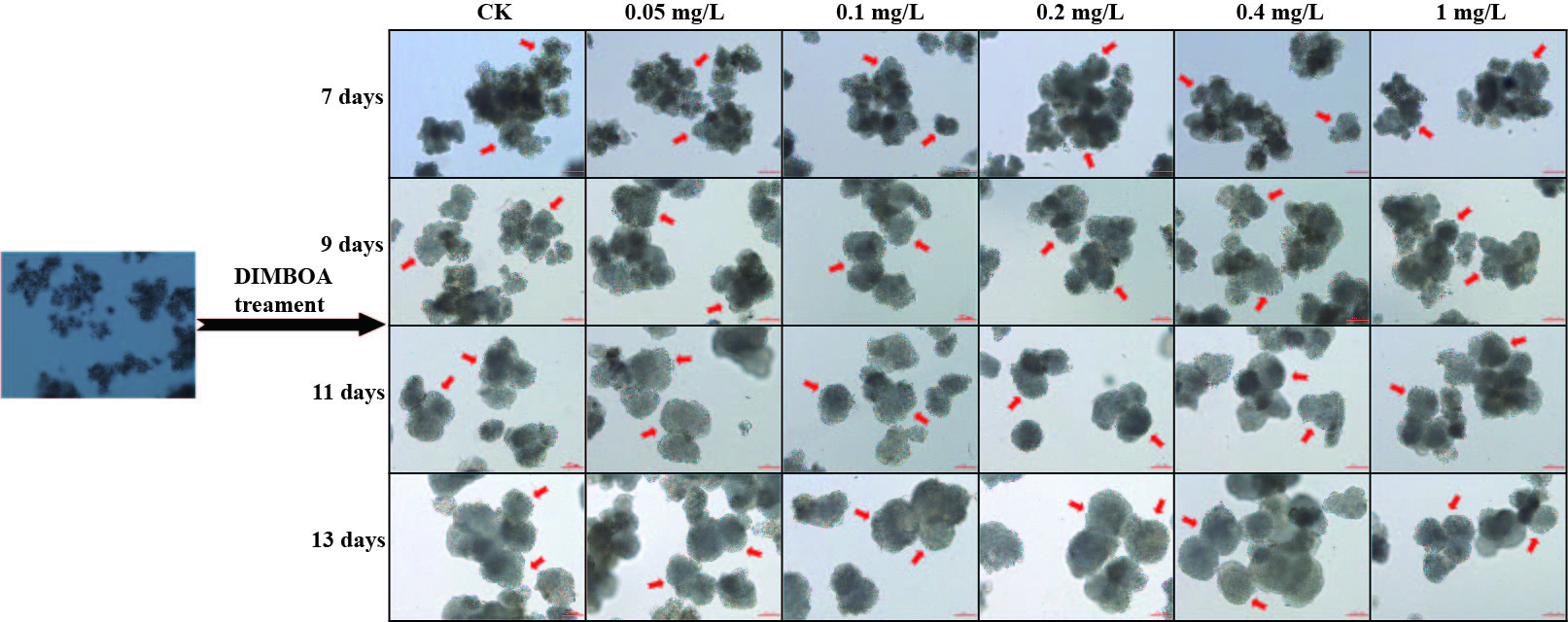

Supplement: Supplementary file 1 [file plants-13-01373-s001.zip › Suppl. Fig S5.jpg]
